# Economic Evaluation of a Multicomponent mHealth Intervention for Stroke Management in Rural China: Cluster-Randomized Trial With 6-Year Follow-Up

**DOI:** 10.2196/75326

**Published:** 2025-09-11

**Authors:** Bolu Yang, Enying Gong, Xingxing Chen, Jie Tan, Nicholas Peoples, Yuhan Li, Jiayu Cai, Yan Li, Brian Oldenburg, Chen Chen, Dejin Dong, Xiaochen Zhang, Eric Finkelstein, Lei Si, Lijing L Yan

**Affiliations:** 1 School of Public Health Wuhan University Wuhan China; 2 Global Health Research Center Duke Kunshan University Kunshan China; 3 School of Population Medicine and Public Health Chinese Academy of Medical Sciences & Peking Union Medical College Beijing China; 4 State Key Laboratory of Respiratory Health and Multimorbidity Chinese Academy of Medical Sciences & Peking Union Medical College Beijing China; 5 Harvard-affiliated Emergency Medicine Residency Mass General Brigham Boston, MA United States; 6 School of Public Health Shanghai Jiao Tong University Shanghai China; 7 Department of Population Health Science and Policy Icahn School of Medicine at Mount Sinai New York, NY United States; 8 Baker Heart and Diabetes Institute Melbourne Australia; 9 La Trobe University Melbourne Australia; 10 Center for Disease Control and Prevention Xingtai Xingtai China; 11 Programme in Health Services & Systems Research Duke-NUS Medical School Outram Singapore; 12 Duke Global Health Institute Duke University Durham, NC United States; 13 Translational Health Research Institute Western Sydney University Penrith Australia; 14 School of Health Sciences Western Sydney University Campbelltown Australia

**Keywords:** economic evaluation, stroke secondary prevention, budget impact analysis, mHealth, cluster-randomized trial

## Abstract

**Background:**

To bridge the gap between clinical guidelines and suboptimal stroke management in rural settings, we conducted an implementation trial using evidence-based, mobile health–enabled strategies to empower primary care providers in rural China. The system-integrated and digital technology–enabled model of care (SINEMA) model was shown to significantly reduce blood pressure and mortality among people with stroke in rural China.

**Objective:**

This study aimed to evaluate the cost-effectiveness of the SINEMA intervention within both the active trial and the post-trial observational periods and its budget impact for potential nationwide scalability.

**Methods:**

In the cluster-randomized implementation trial (the SINEMA trial), 50 villages were randomized to either a 1-year intervention (2017-2018) or usual care, with 1299 patients with stroke followed up until 2022-2023—6 years after the trial baseline. The incremental cost-effectiveness ratios (ICER) for systolic blood pressure reduction and quality-adjusted life year gains were estimated from a health sector perspective. Both probabilistic and deterministic sensitivity analyses were conducted to assess the robustness of the findings. Additionally, a budget impact analysis was performed from a public payer perspective to estimate the per-capita and total costs of national scale-up under 2 scenarios: a standalone intervention and integration into the existing basic public health service system.

**Results:**

The ICER per 1 mmHg systolic blood pressure reduction was $8.4 for the within-trial estimation. The ICER per quality-adjusted life year gained was $837.9 within-trial and $727.9 post-trial, both highly cost-effective relative to any commonly adopted thresholds and robust in sensitivity analyses. The first-year budget impact ranged from $115.6 million to $197.7 million in the 2 scenarios, reducing to $46.6 million to $78.7 million by year 5, with a per-capita cost of $0.03-$0.06.

**Conclusions:**

Our findings demonstrate that the SINEMA intervention was cost-effective during the trial period and remained so throughout the 6-year sustainability observation period. These results highlight the potential of adopting similar health system–integrated, mobile health–enabled strategies to enhance the management of stroke and other chronic diseases in resource-limited settings.

**Trial Registration:**

ClinicalTrials.gov NCT0318585, ClinicalTrials.gov NCT05792618; https://clinicaltrials.gov/study/NCT03185858 and https://clinicaltrials.gov/study/NCT05792618

**International Registered Report Identifier (IRRID):**

RR2-10.3389/fneur.2023.1145562

## Introduction

Stroke is a leading cause of mortality and disability, representing a major global health burden with approximately 4.4 million deaths annually [1]. Beyond mortality, stroke survivors incur substantial direct and indirect economic costs, which impose a significant financial burden on individuals, families, and health care systems [2,3]. In rural China, the number of stroke survivors is higher than in urban areas, with a stroke prevalence of approximately 1291.1 cases per 100,000 individuals, and this number continues to rise, encompassing around 5 million survivors [4]. To address this issue, significant evidence exists regarding management and intervention guidelines for stroke survivors in China [5-7]. However, there is a lack of practical implementation to test the effectiveness of these strategies in preventing stroke recurrence and providing essential care, particularly in resource-constrained settings [8]. To address such unmet health gaps, we developed the system-integrated and technology-enabled model of care (SINEMA), a primary care–based intervention with digital health components, to empower primary health care providers for secondary stroke prevention [9]. Through a cluster-randomized implementation trial, we have shown that the SINEMA intervention significantly reduced systolic blood pressure (SBP), stroke recurrence, hospitalization, and disability at 12 months postbaseline [10]. Follow-up data collected in October 2022 and May 2023 highlighted the sustained effectiveness of the intervention, showing significant SBP reductions and a mortality risk reduction of over 20% [11].

Although the effectiveness of the SINEMA model has been demonstrated through a cluster-randomized implementation trial and long-term follow-up [10,11], guidelines emphasize the importance of recording on-site costs to validate the cost-effectiveness of such intervention trials [12]. For example, 2 studies on poststroke management interventions have not only proven their effectiveness but also demonstrated their cost-effectiveness—although both used different telehealth strategies [13,14]. In resource-limited settings, researchers have applied community-based workforce strategies, similar to the SINEMA model, to manage other chronic diseases such as hypertension [15-17]. While these strategies have shown both effectiveness and cost-effectiveness, they did not integrate mobile health (mHealth) technology, and only 2 studies have conducted long-term post-trial follow-up. Previous studies have demonstrated the increasing use of mHealth interventions in primary care settings for managing chronic diseases in low- and middle-income countries (LMICs) with modest evidence of effectiveness in behavioral and clinical outcomes [18], while the evidence regarding how best to manage individuals with stroke history and the cost-effectiveness of mHealth interventions remains limited [19]. Such gaps raise questions about the cost-effectiveness of integrating mHealth technology into the primary care model and the sustainability of such intervention strategies [20-22].

By leveraging comprehensive data from both the within-trial and long-term post-trial follow-up of the SINEMA trial, this study addresses the evidence gap regarding the cost-effectiveness of an mHealth technology–based primary care model for stroke secondary prevention and its sustained outcomes in resource-limited settings. Additionally, we incorporate budget impact analyses to evaluate the feasibility of scaling up similar approaches that integrate mHealth and community-based workforce strategies. Our findings provide valuable insights and practical support for implementing evidence-based chronic disease management strategies in China and other LMICs facing similar challenges.

## Methods

### SINEMA Trial Design and Participants

This study presents an economic evaluation based on the SINEMA trial, a cluster-randomized implementation trial conducted in a rural region of Hebei Province, China. We conducted both within-trial and post-trial economic evaluations from the health sector perspective, using data from the 1-year trial (ClinicalTrials.gov NCT03185858) and a 6-year observational follow-up postbaseline (ClinicalTrials.gov NCT05792618). Additionally, we performed a budget impact analysis from the public payer perspective.

Detailed information on trial design [9], mHealth intervention design and evaluation [23,24], within-trial results on health outcomes [10], and long-term follow-up health outcomes [11,25] have been published [9,24]. In brief, 50 villages from 5 townships were randomly assigned in a 1:1 ratio to either the intervention or control arm, with stratification by the township. A total of 1299 participants who had been diagnosed with stroke but were in a clinically stable condition were recruited between June 23 and July 21, 2017, and followed up at 12 months. Village doctors underwent training, were equipped with the SINEMA app, and performed monthly follow-up visits for patients with stroke with support from township physicians and financial incentives. Patients with stroke received these visits either at village clinics or at home and were delivered with daily voice messages containing health education on medication adherence and physical activities. In the control arm, village doctors continued their standard practices as usual, involving general clinical care and the delivery of the basic public health services, which included quarterly follow-up visits for some patients with hypertension or diabetes at no out-of-pocket cost [26].

After concluding the 1-year intervention phase, we proceeded with follow-up data collection to assess the long-term effectiveness of the intervention in October 2022 and May 2023 – approximately 5 and 6 years after the trial baseline. The same methodologies were applied with blinded data collectors.

### Study Design of Cost Effectiveness and Budget Impact Analysis

To ensure transparency, we published the study protocol for within-trial economic evaluation [27]. We adhered to the Consolidated Health Economic Evaluation Reporting Standards 2022 guidelines (Table S1 in Multimedia Appendix 2). We describe the previously published within-trial economic evaluation protocol briefly below as well as the post-trial cost-effectiveness analyses and national budget impact analyses in more detail. A CONSORT (Consolidated Standards of Reporting Trails) 2025 checklist is provided in Multimedia Appendix 3.

### Assessment of Costs

Costs were categorized and calculated from the use of health care resources and program delivery costs. Discounting was only applied to post-trial economic analysis, where an annual discount rate of 5% was applied. We adjusted all costs to reflect the 2023 US dollar, using the rural consumer price index for conversion [28].

Costs associated with health care resource use include inpatient care, outpatient care, and medication expenses. For the within-trial period, inpatient costs were retrospectively collected from the admission systems of 4 major hospitals at our study sites, which captured about 98% of inpatient care in the region and were verified against patients’ self-reported history of inpatient care. These records were categorized into 3 groups: stroke-related inpatient care, cardiovascular disease–related inpatient care (excluding stroke), and other causes of inpatient care (Table S2 in Multimedia Appendix 2). For the base case analysis, all-cause inpatient care costs were included in the analysis. As the data on outpatient costs from medical insurance scheme records was not available, we estimated outpatient costs by using the average cost of outpatient visits excluding the medication costs based on existing literature [29].

Medication costs were valued by multiplying the amount of medication used and the unit price for each type of medication. The questionnaires contained questions regarding the specific subtypes of medication used and the duration and adherence of medication use for 3 major types of medications including antiplatelet, statins and antihypertensive drugs (Multimedia Appendix 1). The unit cost of medication was estimated from the municipal government procurement website [30]. Prices for each specific medication were cross-referenced with local village doctors, and the median values for each category of medications were used to ensure balance at baseline. Outpatient costs, excluding medication costs, were estimated based on existing official Chinese statistics [29]. Resources supporting the SINEMA program—including the development of the SINEMA app, compensation for human resources, and voice message delivery fees—were estimated based on detailed project financial records.

The post-trial analysis relied on patients’ self-reported data collected during the 6-year sustainability observation period. Hospitalization costs were estimated using the number of hospital visits recorded in the post-trial period and the average costs associated with different causes of inpatient visits, as determined during the within-trial period. Variables collected during follow-up and the detailed assumptions underlying these estimations are presented in Tables S3 and S4 in Multimedia Appendix 2.

### Assessment of Effectiveness and Utility Outcomes

The within-trial measure of effectiveness is the mean reduction in mmHg in SBP from baseline to the end of follow-up at 1 year after randomization in the SINEMA trial. The health state utilities for the SINEMA study participants were estimated from the EQ-5D-5L using the Chinese EQ-5D-5L value set [31]. The quality-adjusted life years (QALYs) gained for both the within-trial period and post-trial period were calculated using the area under the curve approach [32]. For participants who died during this timeframe, their status and date of death were confirmed using the death registry system. A utility score of 0 was then assigned from the date of death onward. For the post-trial period, we similarly calculated the intervening missing utility values by using a linear imputation method and then summed the QALYs on an annual basis (Table S4 in Multimedia Appendix 2).

### Economic Evaluation and Statistical Analysis

The incremental cost-effectiveness ratios (ICERs) were computed by dividing the mean difference in costs by the mean difference in effectiveness outcomes. ICERs were represented as additional cost per 1 mmHg reduction in SBP and per QALY gained between the SINEMA intervention and usual care.

We used a mixed multilevel effect model to evaluate effectiveness, with a random intercept for the cluster (village) and a fixed effect for townships to account for the stratified design, baseline SBP or baseline utility value, age, and sex based on the intention-to-treat principle. We used bootstrapping techniques to investigate the uncertainty around the ICERs by generating 5000 replications to estimate the combined uncertainty of both cost and health outcomes and plotted on the cost-effectiveness plane to represent the uncertainty from 5000 estimates. To understand the cost-effectiveness of the SINEMA intervention across a spectrum of willingness-to-pay (WTP) thresholds, we used cost-effectiveness acceptability curves [33]. The WTP threshold was set as $18,766, equivalent to 1.5 times China’s gross domestic product per capita in 2023 [34-36].

We performed both probabilistic sensitivity analysis by implementing nonparametric bootstrapping with 5000 replications and deterministic sensitivity analyses by assessing the impact of varying key input parameters individually. This involved evaluating the implications of adapting the 95% CI for health state utilities, considering 50% cost scenarios in both within-trial and post-trial economic evaluations. Additionally, we considered changes in hospitalization costs and each component of intervention to gauge the robustness of our findings. All statistical analyses were conducted using Stata version 17.

### Budget Impact Analysis

We conducted the base-case budget impact analysis from a public payer perspective, quantifying all costs associated with the SINEMA program. These costs include the following: (1) compensation for township physicians, (2) compensation for the local project manager, (3) compensation for village doctors, (4) training costs, (5) voice messaging costs, (6) annual system maintenance and management costs, and (7) intervention development costs. The budget impact analysis assumes that the SINEMA program would be scaled up using existing village doctors and reach all rural households within the first year of implementation.

To quantify total costs, we estimated both unit costs and the number of people expected to receive SINEMA. We first assumed SINEMA as a standalone project as scenario A. Since a large proportion of patients with stroke already receive nationwide services under the national basic public health services, which involves quarterly visits by village doctors to monitor blood pressure, we integrated the SINEMA program into the current public health services for scenario B analysis. The estimation method for costs and the eligible population are detailed in Table S5 in Multimedia Appendix 2. The health service utilization analysis showed that SINEMA led to a significant and consistent increase in the use of antihypertensive and antiplatelet medications compared to usual care. Therefore, these medication costs are included (Table S6 in Multimedia Appendix 2). We assumed that supervision and system coordination costs would decline after the first year, reflecting typical scale-up patterns observed in similar community-based interventions [37,38], where oversight and training expenses declined substantially as programs became integrated into routine service delivery (Table S5 in Multimedia Appendix 2).

### Ethical Considerations

The study protocol was approved by the Institutional Review Boards of Duke University (Pro00082130), Beijing Tiantan Hospital (2015MRC0012), and Duke Kunshan University (China). All participants, including health care providers and patients, provided written informed consent after being informed of the study’s purpose, procedures, potential benefits and risks, and data confidentiality safeguards. In addition, cluster-level consent was obtained from township and village opinion leaders. This post-trial follow-up study was approved by the ethical boards of the Chinese Academy of Medical Sciences (CAMS&PUMC-IEC-2022-062 and CAMS&PUMC-IEC-2024-047) and Duke University (Pro00082130-AMD-9.1).

## Results

**Figure 1:**
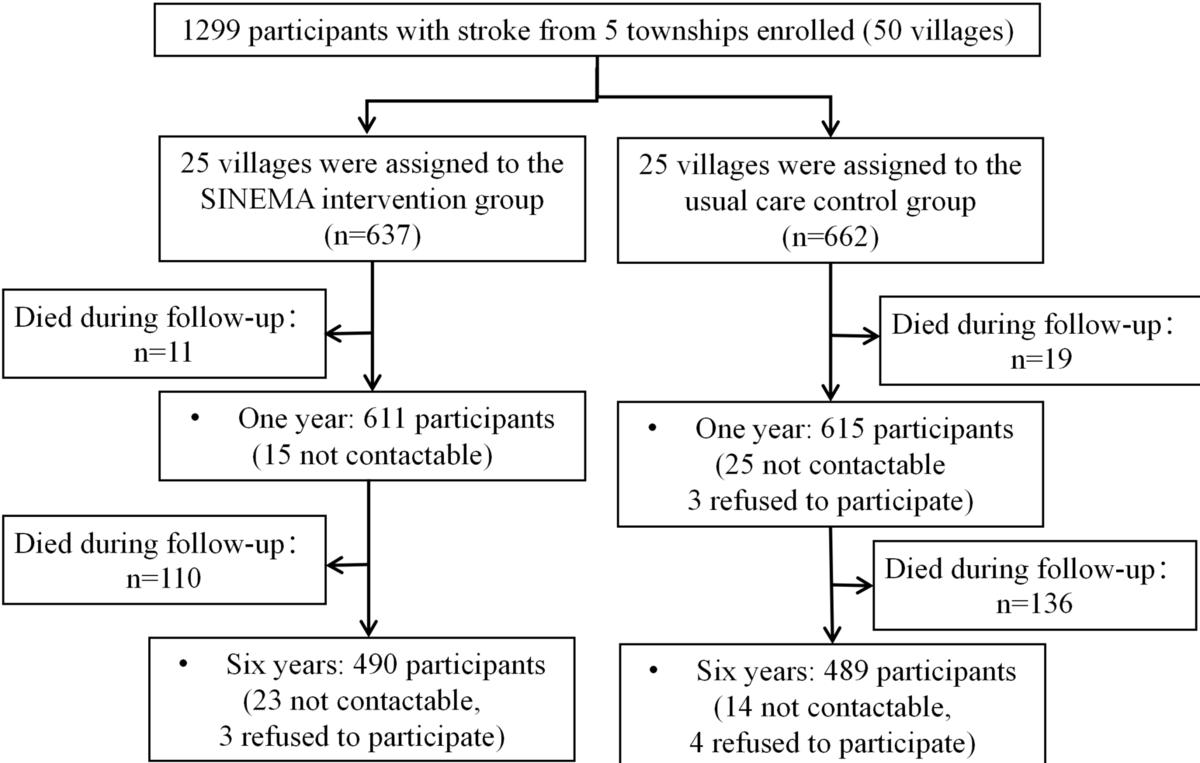
CONSORT (Consolidated Standards of Reporting Trails) flowchart of the SINEMA trial and the post-trial follow-up.

Both groups had comparable baseline characteristics (Table S7 in Multimedia Appendix 2). Participants’ average age was 65.6 years (SD 8.2), with 71.2% (n=925) having no or only primary education. Their average SBP was 145.9 mm Hg (SD 22.4), and the mean health state utility score was 0.80 (SD 0.20). By the 1-year follow-up, 30 out of 1299 participants (2%) died, and 43 out of 1299 participants (3%) were lost to follow-up, leaving 611 and 615 participants in the intervention and control groups, respectively, who completed the survey (Figure 1 and Table S8 in Multimedia Appendix 2).

### Within-Trial Cost-Effectiveness Analysis

The total program implementation costs were $32,389.2, averaging $50.8 per participant in the intervention group. During the trial period, the average total cost per person for inpatient visits was slightly higher in the control arm, though the difference was not statistically significant (mean difference: –$32.4, 95% CI –$107.3 to $172.1). The costs for antiplatelets and antihypertensives were significantly higher in the intervention arm, with mean differences of $2.11 (95% CI $1.27-$2.98) and $2.99 (95% CI $0.71-$5.27), respectively, suggesting an increased use of these medications. The total costs, including both intervention-related costs and health service use, were $236.5 per participant in the intervention arm and $213.1 in the control arm, resulting in a mean cost difference of $23.3 (95% CI: –$115.5 to $162.0). The bootstrapped analysis showed a mean difference of –2.8 mmHg (95% CI –4.7 to –1.0) in SBP and a 0.03 (95% CI 0.02-0.04) difference in QALYs between the 2 arms (Tables 1 and 2).

**Table 1:** Intervention costs during the within-trial period.

Intervention costs	Unit cost ($)	Quantity	Total costs ($)	Total cost per participant ($)
Compensation for township physicians	228.9	5	1144.7	1.8
Compensation for village doctors	414.8	25	10369.9	16.3
Compensation for local project manager	740.7	1	740.7	1.2
Compensation for county specialists	134.7	2	269.3	0.4
Voice messages	0.02	97,000	1548.8	2.4
Printing materials	0.5	700	336.7	0.5
System maintenance and management costs per year	1481.4	1	1481.4	2.3
Intervention development fee	16,497.6	1	16,497.6	25.9
Total	N/A^a^	N/A	32,389.2	50.8

^a^Not applicable.

**Table 2:** Health service utilization costs and effectiveness during the within-trial period.

Category				Intervention, mean (SD)^a^		Control, mean(SD)^a^		Mean difference (95% CI)
**Health service utilization costs ($)^b^**								
	Outpatient visit (excluding medication costs, per participant)		45.5		45.5		0	
	All causes of inpatient visit (per participant)^c^		218.3		254.5		–2.4 (–107.3 to 172.1)	
	**Medication costs (individual adherence discounted)^d^**							
		Antiplatelet	11.0		8.9		2.11 (1.27 to 2.98)	
		Statin	3.2		3.1		0.09 (–0.67 to 0.50)	
		Antihypertensive medicines	23.1		20.3		2.99 (0.71 to 5.27)	
	Average total costs^e^		236.5		213.1		23.3 (–115.5 to 162.0)	
**Effectiveness**								
	Change in systolic blood pressure (mmHg)^f^		–7.1 (0.6)		–4.3 (0.7)		–2.8 (–4.7 to –1.0)	
	Change in health-related quality of life score^f^		0.01 (0.01)		–0.06 (0.01)		0.06 (0.04 to 0.08)	
	Average quality-adjusted life years gained		0.81 (0.19)		0.79 (0.20)		0.03 (0.02 to 0.04)	

**^a^**Cost values represent mean costs per participant calculated from aggregated data, not individual-level distributions; therefore, SDs are not reported.

^b^Health service utilization costs are reported as total costs per participant.

^c^Inpatient costs represent the mean total costs among participants who had at least one hospitalization.

^d^Medication costs represent the mean total costs among participants who used medication.

^e^The total cost reflects the mean cost per participant across the entire study population, including those without any hospitalization or medication use.

^f^Change represents the difference from baseline to 1-year follow-up within the trial period.

**Figure 2:**
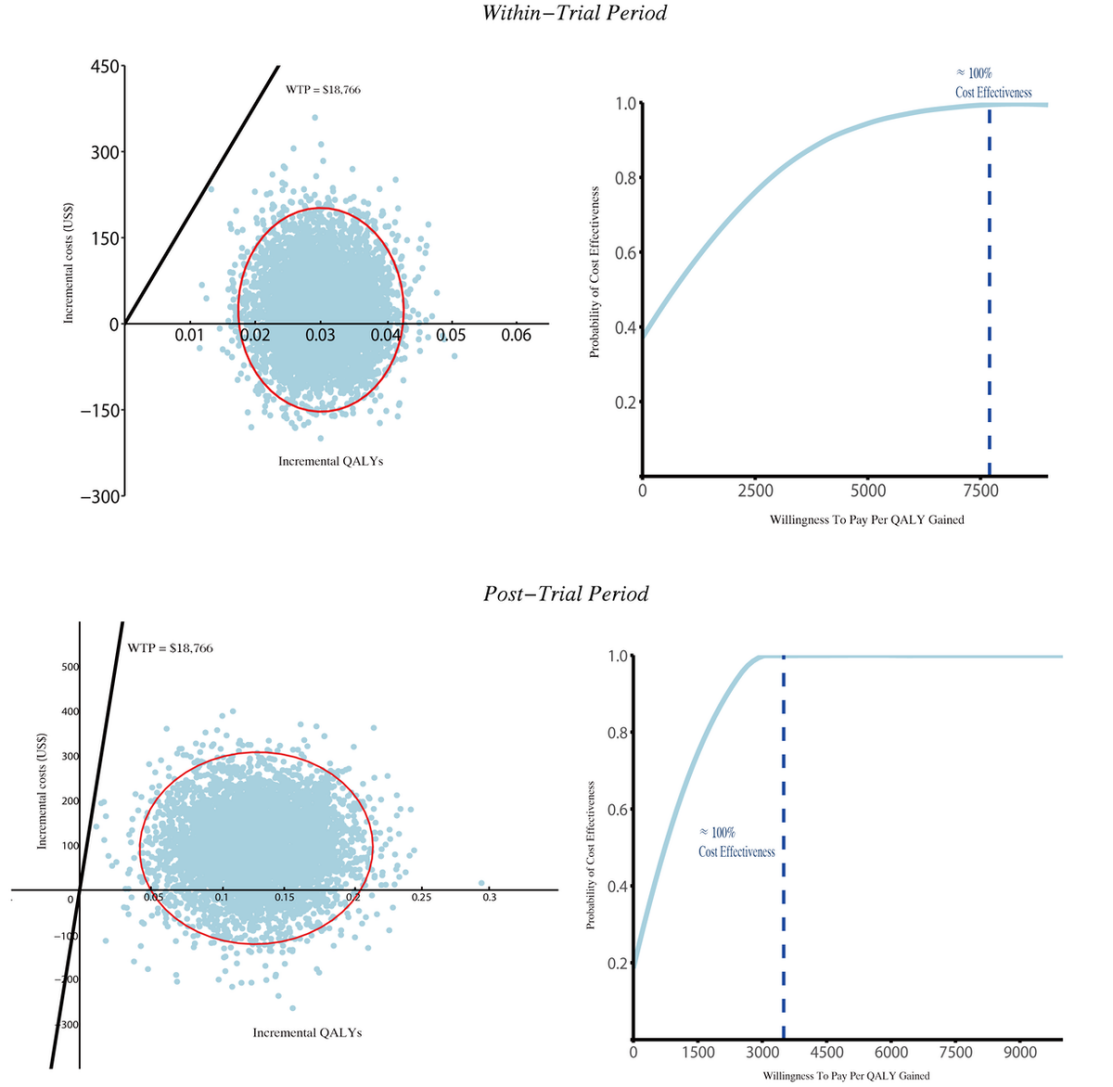
All currency values are in 2023 US$. ICER: incremental cost-effectiveness ratio. “Within-trial period” refers to the intervention phase (2017–2018). “Post-trial period” refers to the time after the intervention concluded (from 2018 onward). Each blue dot represents an outcome from one of 5,000 iterations. The red circle indicates the 95% confidence interval (CI) of these outcomes. The dashed vertical line marks the probability of cost-effectiveness across willingness-to-pay thresholds, including the estimated threshold at which cost-effectiveness reaches 100%.

The ICER was $8.4 per mmHg reduction in SBP (95% CI 4.3-12.6) and $837.9 per QALY gained (95% CI $766.9-$908.9). The bootstrapped estimates of the mean differences in costs and effects were concentrated in the northeast quadrant of the cost-effectiveness plane (Figure 2, top left panel), indicating that the SINEMA intervention had a 100% probability (5000/5000 replications) of being cost-effective based on the predefined WTP threshold of $18,766. The cost-effectiveness acceptability curves for QALYs demonstrated that the probability of SINEMA being cost-effective was 100% at a WTP of approximately $7700 (Figure 2, top right panel).

The scenario analysis of the ICER to changes in key parameters is presented in Table 3. The base case analysis estimated an ICER of $8.4 (95% CI $4.3-$12.6) per unit reduction in SBP and $837.9 (95% CI $766.9-$908.9) per QALY gained during the trial. Scenario analyses revealed that the ICER ranged from $3.8 to $23.0 per mmHg reduction in SBP and from $298.7 to $1363.9 per QALY gained across different adjustment models. Among intervention-related cost components, variations in the compensation for village doctors had the largest influence on the ICER. Regarding health service utilization costs, changes in annual hospitalization costs contributed the most to variations in the ICER.

**Table 3:** Sensitivity analyses for calculating incremental cost-effectiveness ratios during the within-trial period.

Additional scenario analysis			Range of values^a^		Cost per mmHg SBP^b^ change^c^ ($)					Cost per QALY^d^ gained^c^ ($)			
					Low	High			Low		High		
**Changes in effectiveness-related parameters**													
	Systolic blood pressure reduction, mmHg^e^	1.0-4.7		5.2			23.0		N/A^f^			N/A	
	Difference in QALYs between 2 groups^e^	0.02-0.04		N/A			N/A		603.4			1206.8	
	Incremental mean medication adherence	0.01-0.04		6.3			8.6		694.1			942.7	
**Changes in cost-related parameters ($)**													
	Compensation for township physicians^g^	572.4-1717.1		8.1			8.8		805.5			870.3	
	Compensation for village doctors^g^	5194.9-15554.9		5.4			11.2		539.4			1114.8	
	Compensation for local project manager	370.4-1111.1		8.2			8.5		812.7			855.9	
	Compensation for county specialists	134.65-403.95		8.3			8.4		823.5			837.9	
	Voice messages	774.4-2323.2		8.1			8.8		805.5			873.9	
	Printing materials	168.35-505.05		8.4			8.4		834.3			837.9	
	System maintenance and management costs per year	740.7-2222.1		7.9			8.7		784.0			866.7	
	Intervention development fee	8248.8-24,746.4		3.8			13.0		381.2			1294.6	
	Incremental annual medication cost	2.6-7.8		7.5			12.9		692.6			975.1	
	Incremental annual hospitalization cost	–48.6 to 16.2		4.8			16.7		298.7			1363.9	

^a^The range was defined as a 50% decrease or increase from the base case for key parameters, except for systolic blood pressure reduction and difference in QALYs between 2 groups.

^b^SBP: systolic blood pressure.

^c^Calculated based on the 5000 bootstrapping and adjusted for baseline outcome, township, sex, and age.

^d^QALY: quality-adjusted life year.

^e^Range values are the bounds of the 95% CIs from the primary outcome analysis in the SINEMA trial.

^f^Not applicable.

^g^Presented as the average cost for township physicians and village doctors including performance-based incentives.

### Post-Trial Cost-Effectiveness Analyses

During the 6-year sustainability observation period, 246 deaths were observed (intervention group: 110/626, 17.3%; control group: 136/643, 20.4%). Of the 1023 surviving participants, 979 (95.7%) completed the survey and 44 (4.3%) were lost to follow-up (Figure 1 and Table S8 in Multimedia Appendix 2).

The total post-trial costs were higher in the intervention group, with a mean difference of $91.5 (95% CI –$67.9 to $250.1). The mean difference in QALYs between the groups was 0.13 (0.06-0.20). This led to a lower ICER of $727.9 (95% CI $626.4-$829.4) in the post-trial period compared to the within-trial period (Table 4). The bootstrapped estimates demonstrated that the probability of the SINEMA intervention being cost-effective remained at 100% (Figure 2, bottom left panel). The cost-effectiveness acceptability curve indicates that the willingness-to-pay threshold for 100% cost-effectiveness is approximately $3,000 (Figure 2, bottom right panel). Scenario analysis revealed that the ICER varied the most when hospitalization costs were adjusted at the second follow-up, but no parameter changes affected the overall cost-effectiveness conclusions (Table 5).

**Table 4:** Costs and effectiveness during the post-trial period.

Category				Intervention, mean (SD)^a^		Control, mean (SD)^a^		Mean difference (95% CI)
**Costs ($)**								
	Outpatient visit (excluding medication costs, per participant)			124.2		122.7		1.74 (–0.68 to 3.63)
	All cause of inpatient visit (per episode)			639.8		564.9		74.80 (–97.96 to 247.49)
	**Medication costs (individual adherence discounted)**							
		Antiplatelet	52.7		44.3		8.41 (3.56 to 13.25)	
		Statin	12.0		11.4		0.53 (–1.25 to 2.31)	
		Antihypertensive medicines	56.7		48.0		8.69 (1.87 to 15.50)	
	Average total costs			889.9		798.4		91.50 (–67.87 to 250.80)
**Effectiveness**								
		Change in health-related quality of life score^b^	–0.24 (0.01)		–0.23 (0.01)		–0.02 (–0.06 to 0.02)	
		Average Quality Adjusted Life Years Gained	2.51 (0.03)		2.41 (0.03)		0.13 (0.06 to 0.20)	

^a^Cost values represent mean costs per participant calculated from aggregated data, not individual-level distributions; therefore, SDs are not reported.

^b^Change represents the difference from 1-year to 4.5-year follow-up in the post-trial period.

**Table 5:** Sensitivity analyses for calculating incremental cost-effectiveness ratios during the post-trial period.

Additional scenario analysis		Range of values^a^	Cost per QALY^b^ gained^c^ ($)	
			Low	High
**Changes in cost-related parameters**				
	Medication cost at the second follow-up ($)	82.7-160.2	717.6	843.1
	Hospitalization cost at the second follow-up ($)	391-994.5	219.2	1328.8
	Discount (%)	3-8	511	860.5
**Changes in effectiveness-related parameters**				
	Difference in QALYs between 2 groups^d^	0.07-0.19	488.8	1325.8
	Incremental mean medication adherence^d^	0.47-1.41	660.5	839.7

^a^The value range was defined as ±50% of the base case for key parameters, except for the difference in QALYs between the 2 groups and the incremental mean medication adherence.

^b^QALY: quality-adjusted life year.

^c^Calculated based on the 5000 bootstrapping and adjusted for baseline outcome, township, sex, and age.

^d^Low and high values are the bounds of the 95% CIs from the primary outcome analysis in the SINEMA trial.

### Budget Impact Analysis

Based on a stroke prevalence of 2.58% [39] and a rural proportion of patients with stroke of 52.9% [29], the eligible population for scaling up the SINEMA intervention to rural China was estimated to be 11,676,010 people. For subsequent years, we estimated the eligible population based on an annual stroke incidence rate of 8.26% [39], assuming a 1% annual national population growth and a 0.16% death rate [29]. This broader reach enabled the amortization of fixed costs such as system development and oversight across a much larger population, resulting in a reduced per capita cost. In scenario A, the program cost was estimated at $16.93 per participant, leading to a total budget impact of $197.72 million in the first year. Since intervention costs were only incurred in the first year, expenditure decreased in subsequent years. By the fifth year, with 4 visits from village doctors per year maintained, the expenditure decreased slightly by 3.2%, reaching $78.07 million (Table 6).

**Table 6:** Budget impact analysis of the system-integrated and digital technology–enabled model of care (SINEMA) intervention in year 1 and year 5.

Item			Scenario A: stand-alone project					Scenario B: integrated with NBPH^a^		
			Year 1		Year 5		Year 1			Year 5
**Eligible population, n**			11,676,010		11,695,170		11,676,010			11,695,170
	Eligible participants receiving NBPH^b^	N/A^c^		N/A		7,125,158			7,136,851	
	Eligible participants excluded from NBPH^b^	N/A		N/A		4,550,852			4,558,319	
Cost per participant ($)			16.93		6.68		9.90			3.98
Total budget impact^d^ ($)			197,726,357		78,067,406		115,585,636			46,592,046
Total population, n			1,411,750,000		1,459,785,833		1,411,750,000			1,459,785,833
Cost per capita^e^ ($)			0.14		0.06		0.08			0.03

^a^NBPH: national basic public health.

^b^Eligible participants receiving NBPH are individuals with stroke and hypertension or diabetes living in rural communities. Eligible participants excluded from NBPH are individuals with stroke who do not have hypertension or diabetes.

^c^Not applicable.

^d^Total budget impact is rounded to the nearest $10.

^e^Calculated as the total budget impact divided by the total population.

In scenario B, where the SINEMA intervention was integrated into the existing national basic public health services, we identified that 7,125,158 participants—approximately 61% of the eligible population—had already received these services [39]. The cost per participant in this scenario was reduced to $9.90, which resulted in a total budget impact of $115.59 million in the first year, only slightly over half of the costs in scenario A **(**Table 6). Results for all years and specific components are shown in Table S9 in Multimedia Appendix 2. Compensation for village doctor visits accounted for the largest portion of the total budget in both scenarios.

## Discussion

### Principal Results

With a mean follow-up of 6 years of the SINEMA trial, we conducted within-trial and post-trial cost-effectiveness analyses and budget impact analyses for the SINEMA model—an integrated mHealth primary care model for stroke management. The hybrid, type 2, effectiveness-implementation SINEMA trial has previously demonstrated not only the effectiveness of evidence-based strategies but also the feasibility of their implementation strategies [10,40]. Building on these findings, this study further showed that the SINEMA model was cost-effective for controlling blood pressure and improving QALYs compared to usual care, both within the 12-month trial period and during the 5-year post-trial follow-up. The base case analysis estimated an ICER of $8.4 per unit reduction in SBP, $837.9 per QALY gained during the trial, and $727.9 per QALY gained in the post-trial period, much lower than the WTP thresholds. Sensitivity analyses across various scenarios consistently supported these findings, reinforcing the robustness of the results. The budget impact analysis further supported the potential of the SINEMA intervention as a sustainable solution that incorporates both system-level consideration and mHealth empowerment, suitable for national scale-up in managing stroke and potentially for other chronic diseases.

### Comparison With Prior Work

Our results demonstrating the high cost-effectiveness of the SINEMA model highlighted the importance of investing in primary health care and mHealth technology for stroke management and improving patient outcomes. To our knowledge, no cost-effectiveness studies on stroke interventions using similar mHealth technology have been conducted in LMICs, aside from one study from the United States and another from India, both of which used telemedicine-based approaches [13,14]. In a US-based study, the ACCESS teleneurology program for acute stroke care reduced unnecessary patient transfers and improved early treatment, resulting in a cost saving of $4241 and a QALY gain of 0.20 per patient over a 90-day horizon, demonstrating economic dominance over standard care [14]. In the Indian study involving 1200 postneurosurgery patients, telemedicine follow-up care was more effective and less costly than routine in-person care, with a cost per utility unit of $40.5 versus $105.4 and an ICER of –$537 [13]. However, the large differences in intervention design and context preclude direct comparison with our results. Moreover, telemedicine adoption in rural China has faced significant barriers, particularly related to digital literacy [41]. The SINEMA model, which includes voice message–based health communication, may help improve digital health acceptance among rural populations. Its cost-effectiveness has been clearly demonstrated, with a cost of $837.9 per QALY gained during the within-trial period—well below the internal cost-effectiveness threshold in China (eg, 1.5 times the 2023 gross domestic product per capita of $18,766).

We also compared our results with other community-based blood pressure–lowering trials in LMICs in rural Asia. For example, the COBRA trial in Pakistan reported an ICER of $23 per mmHg reduction in SBP [42]. The COBIN intervention in Nepal, though initially cost-effective [37], saw a puzzling reversal of its benefits over time from a 5-mmHg reduction at 1 year to a 5-mmHg increase by 5 years [21]. In contrast, our 5-year post-trial results demonstrated sustained effectiveness of the intervention over time [11]. The ICER in our study was $10.5 per mmHg reduction in SBP, which was lower than that in the COBRA trial. One possible explanation for the higher and sustained long-term cost-effectiveness observed in our study, which was not accounted for in their analyses, could be the integration of mHealth technology, including a provider-facing Android app and patient-oriented voice messages.

Based on our budget impact analysis, scaling up the SINEMA model nationwide is both affordable and impactful. In China, while per capita health expenditure surged from RMB 1090 to RMB 4700 (US $164.9 to US $710.3) [43], spending on specialized public health organizations increased in absolute terms but dropped as a share of total health expenditure, from 8.58% to 5.47% [44]. In our study, the required investment for scaling up the SINEMA intervention represents about 0.15% of the basic national health services expenditure and could reduce hospitalization costs, underscoring the importance of investing in primary care and the value of task-shifting strategies from specialists to primary care providers [45,46]. Our study also explored the scenario of integrating the SINEMA app into the national basic public health service, adding intervention components of voice messaging and frequent SBP electronic records, which is estimated at nearly half the costs of the standard intervention. This finding highlighted the potential for saving costs when leveraging mHealth technology within the existing system. However, potential barriers and challenges, such as regional disparities in health care infrastructure, workforce capacity, and digital literacy, may be raised when considering large-scale implementation, which may lead to additional intervention components and additional costs. Furthermore, given that incentives for village doctors consistently accounted for the largest proportion of overall costs, future studies could further explore the strategies of a risk-based, adaptive delivery model that tailors follow-up intensity based on patients’ clinical stability.

### Limitations

Unlike most within-trial or modelling studies, this economic evaluation study based on the SINEMA trial has 3 parts—within-trial and post-trial cost-effectiveness analyses and budget impact analyses. We used extensive data on costs and effectiveness at the individual level collected mainly from our trial data, including mortality for both periods, alleviating the need to rely on blood pressure–based parameter estimates from literature and risk prediction models. Our study has some limitations. First, data on outpatient care were unavailable from the trial, requiring us to use external sources. Nonetheless, outpatient costs constitute only a small proportion of total medical costs. Second, while we collected patient-level data for the past year at the end of the active trial in 2018 and in the 5-year post-trial survey (2022-2023), data during the intermediate years (2019-2021) were missing. We extrapolated data for these years from available data. Extensive sensitivity analyses, varying each parameter by ±50%, found that the results were robust, mitigating concerns about missing data. Third, this trial was conducted in a single city in rural China, which may limit its generalizability. Nonetheless, our budget impact analysis explored the potential for national scale-up and highlighted that this intervention aligns well with the existing health system. The mHealth-enabled system, aligned with the current basic public health services framework, has the potential to enhance the management of stroke and other chronic diseases on a larger scale. Lastly, in the budget impact analyses, we lacked cost data from rural areas beyond the trial site and therefore assumed that intervention costs in other rural settings would be comparable to those observed in the trial, which was conducted in a single rural location. Additionally, we projected potential cost reductions in subsequent scale-up years, particularly related to human resources and the mHealth system. While such assumptions are commonly used in similar budget impact analyses, they remain strong and are inherently difficult to validate. Nonetheless, the analyses offer concrete estimates of potential financial implications and serve as a valuable tool for informing policy decisions and guiding resource allocation.

### Conclusions

In summary, our findings on the cost-effectiveness of the SINEMA model highlight the value of investing in primary care services and mHealth technology for stroke care in rural China. The budget impact analyses provide evidence for sound policymaking and reveal their significant potential for nationwide scaling up in China. While this study was conducted among patients with stroke in a resource-limited region of China, its high cost-effectiveness highlights the need for future research to explore whether and how similar models—integrating mHealth technologies with the primary care workforce—can be adapted for other chronic conditions and comparable settings in LMICs.

## Appendix

Multimedia Appendix 1 Trial protocol and long-term follow-up protocol.

Multimedia Appendix 2 Supplemental tables.

Multimedia Appendix 3 CONSORT checklist.

## Abbreviations

CONSORT: Consolidated Standards of Reporting Trails
ICER: incremental cost-effectiveness ratio
LMIC: low- and middle-income country
mHealth: mobile health
QALY: quality-adjusted Life Year
SINEMA: system-integrated and digital technology–enabled model of care
SBP: systolic blood pressure
WTP: willingness-to-pay

## Data Availability

Deidentified individual data can be made available upon written, detailed request directed to the corresponding author. Requests will be reviewed on the basis of scientific merit, ethical review, legal issues, and regulatory requirements.
