# Supplementary material for: Economic Evaluation of a Multicomponent mHealth Intervention for Stroke Management in Rural China: Cluster-Randomized Trial With 6-Year Follow-Up
Source: JMIR Mhealth Uhealth. 2025 Sep 11;13:e75326. doi: 10.2196/75326 (PMC12464504; doi:10.2196/75326)
Supplement: Multimedia Appendix 2 [file mhealth_v13i1e75326_app2.docx]

**Multimedia appendix**

**Table of Contents**

[**Table S1: CHEERS 2022 Checklist** 2](#_Toc206402708)

[**Table S2: Causes of Hospitalizations** 4](#_Toc206402709)

[**Table S3: Variables Collected in Three Different Time Points** 6](#_Toc206402710)

[**Table S4: Detailed Method for 6-year post-trial baseline effectiveness and cost calculation** 7](#_Toc206402711)

[**Table S5: Scale-up population and cost estimation methodology** 8](#_Toc206402712)

[**Table S6: Impact of the SINEMA Intervention on Health Outcomes** 11](#_Toc206402713)

[**Table S7: Characteristics of participants at baseline refer to the work from Yan et al. ^a^** 12](#_Toc206402714)

[**Table S8: Analysis of Loss to Follow-Up Differences Between Groups Within and Post Trial ^a^** 13](#_Toc206402715)

[**Table S9: Budget impact in China in years 1–5 from the health ministry perspective** 14](#_Toc206402716)

[**Supplementary references** 16](#_Toc206402717)

## **Table S1: CHEERS 2022 Checklist**

|  | **Item** | **Guidance for Reporting** | **Reported in section** |
| --- | --- | --- | --- |
| **TITLE** | | |  |
| Title | 1 | Identify the study as an economic evaluation and specify the interventions being compared. | Page 1  Line 1-3 |
| **ABSTRACT** | | |  |
| Abstract | 2 | Provide a structured summary that highlights context, key methods, results and alternative analyses. | Page 3  Line 42-67 |
| **INTRODUCTION** | | |  |
| Background and objectives | 3 | Give the context for the study, the study question and its practical relevance for decision making in policy or practice. | Page 4-5  Line 71-109 |
| **METHODS** | | |  |
| Health economic  analysis plan | 4 | Indicate whether a health economic analysis plan was developed and  where available. | Page 6  Line 135  Published Study Protocol |
| Study population | 5 | Describe characteristics of the study population (such as age range, demographics, socioeconomic, or clinical characteristics). | Page 9 Line 226-231 and **Supplementary Table 7** |
| Setting and location | 6 | Provide relevant contextual information that may influence findings. | Page 5-6 Line 113-124 |
| Comparators | 7 | Describe the interventions or strategies being compared and why chosen. | Page 5-6 Line 125-130 |
| Perspective | 8 | State the perspective(s) adopted by the study and why chosen. | Page 5 Line 110-115 |
| Time horizon | 9 | State the time horizon for the study and why appropriate. | Page 5 Line 114-118 |
| Discount rate | 10 | Report the discount rate(s) and reason chosen. | Page 6 Line 142-144 |
| Selection of outcomes | 11 | Describe what outcomes were used as the measure(s) of benefit(s) and harm(s). | Page 7 Line 166-175 |
| Measurement of outcomes | 12 | Describe how outcomes used to capture benefit(s) and harm(s) were measured. | Page 7 Line 170-178;  Supplementary file 1: Trial and long-term follow up protocol |
| Valuation of outcomes | 13 | Describe the population and methods used to measure and value outcomes. | Page 7 Line 166-175 |
| Measurement and valuation of resources  and costs | 14 | Describe how costs were valued. | Page 6-7 Line 140-168 |
| Currency, price date, and conversion | 15 | Report the dates of the estimated resource quantities and unit costs, plus the currency and year of conversion. | Page 6 Line 141-144 |
| Rationale and  description of model | 16 | If modelling is used, describe in detail and why used. Report if the model  is publicly available and where it can be accessed. | NA |
| Analytics and assumptions | 17 | Describe any methods for analyzing or statistically transforming data, any extrapolation methods, and approaches for validating any model used. | **Supplementary Table 4 and Supplementary Table 5** |
| Characterizing heterogeneity | 18 | Describe any methods used for estimating how the results of the study vary for sub-groups. | NA |
| Characterizing  distributional effects | 19 | Describe how impacts are distributed across different individuals  or adjustments made to reflect priority populations. | NA |
| Characterizing uncertainty | 20 | Describe methods to characterize any sources of uncertainty in the analysis. | Page 8 Line 192-197 |
| Approach to engagement with patients and others affected by the study | 21 | Describe any approaches to engage patients or service recipients, the general public, communities, or stakeholders (e.g., clinicians or payers) in the design of the study. | Page 7 Line 161-163  Table 1 |
| **RESULTS** | | |  |
| Study parameters | 22 | Report all analytic inputs (e.g., values, ranges, references) including uncertainty or distributional assumptions. | Page 9-14  (Table 1-Table 3) |
| Summary of main results | 23 | Report the mean values for the main categories of costs and outcomes of interest and summarize them in the most appropriate overall measure. | Table 1-Table 3 |
| Effect of uncertainty | 24 | Describe how uncertainty about analytic judgments, inputs, or projections  affect findings. Report the effect of choice of discount rate and time horizon, if applicable. | Figure 1 and Table 3  Line 260-265; 288-290 |
| Effect of engagement with patients and others affected by the study | 25 | Report on any difference patient/service recipient, general public, community, or stakeholder involvement made to the approach or findings of the study | NA |
| **DISCUSSION** | | |  |
| Study findings, limitations, generalizability, and current knowledge | 26 | Report key findings, limitations, ethical or equity considerations not captured, and how these could impact patients, policy, or practice. | Page 16-19 |
| **OTHER RELEVANT INFORMATION** | | | |
| Source of funding | 27 | Describe how the study was funded and any role of the funder in the identification, design, conduct, and reporting of the analysis | Page 20  Line 432-437 |
| Conflicts of interest | 28 | Report authors conflicts of interest according to journal or International Committee of Medical Journal Editors requirements. | Page 20 Line 443 |

NA: Not Applicable

Husereau D, Drummond M, Augustovski F, de Bekker-Grob E, Briggs AH, Carswell C, Caulley L, Chaiyakunapruk N, Greenberg D, Loder E, Mauskopf J, Mullins CD, Petrou S, Pwu RF, Staniszewska S; CHEERS 2022 ISPOR Good Research Practices Task Force. Consolidated Health Economic Evaluation Reporting Standards 2022 (CHEERS 2022) Statement: Updated Reporting Guidance for Health Economic Evaluations. BMJ. 2022;376:e067975.

## **Table S2: Causes of Hospitalizations**

| **Causes of hospitalization** | **Disease type (ICD-10)** ***** |
| --- | --- |
| **Stroke-related diseases** | Stroke-like episode (G45.8)  Reversible ischemic neurological deficit (G45.8)  Reversible ischemic neurological impairment (G45.8)  Posterior circulation ischemia (G45.8)  Basal ganglia hemorrhage (I61.2)  Cerebral artery occlusion (I63.9)  Transient ischemic attack (G45.9)  Cerebral hemorrhage (I61.9)  Sequelae of cerebral hemorrhage (I69.1)  Sequelae of cerebrovascular accident (I69.4)  Cerebral embolism (I63.3)  Cerebral infarction (I63.9)  Sequelae of cerebral infarction (I69.3)  Lacunar infarction (I63.8)  Insufficient cerebral blood supply (G45.9)  Subarachnoid hemorrhage (I60.9) |
| **Cardiovascular diseases (excluding stroke)** | Coronary heart disease (I25.1) - Includes coronary artery atherosclerotic heart disease with arrhythmia and angina pectoris  Heart failure (I50.9) - Includes New York Heart Association classification and acute heart failure  Palpitations (R00.2)  Hypertensive disease (I10) |
| **Other diseases** | Type 2 diabetes mellitus (E11.9)  Hypoglycemic reaction (E16.1)  Vomiting (R11.0) - Including cases to be investigated  Dizziness (R42.9)  Parkinson's syndrome (G20)  Impaired consciousness (R40.4)  Chronic kidney disease stage 1 (N18.1)  Chronic kidney disease stage 5 (N18.5) - Includes chronic renal failure  Electrolyte imbalance (E87.8) - Includes electrolyte disorder  Seizure (G40.9) - Including epilepsy and secondary epilepsy  Renal failure (N19)  Alzheimer's disease (G30.9)  All cardiovascular and cerebrovascular-related diseases  Type 2 respiratory failure (J96.1)  Degenerative knee joint disease (M17.9)  Rheumatoid arthritis (M05.9)  Senile cataract (mature stage) (H25.1)  Osteoporosis (M81.9)  Pulmonary infection (J18.9)  Hematuria to be investigated (R31.9)  Left popliteal cyst (M71.40)  Abdominal discomfort (R10.84)  Gastrointestinal bleeding (K92.2)  Hemorrhagic shock (R57.1)  Severe anemia (D64.9)  Pulmonary embolism (I26.90)  Urethral stricture (N35.1)  Multiple muscle strains (S39.8)  Chronic obstructive pulmonary disease exacerbation (J44.1)  Gallstones (K80.1)  Hydrocele (N43.3)  Cystitis (N30.9)  Cervical disc herniation (M50.1) |

*The categorization of diseases is determined according to the ICD-10 classification

## **Table S3: Variables Collected in Three Different Time Points**

| **Variables collected are in the following categories** | **Collected at baseline** | **Collected at 1-year follow-up** | **Collected at 6-year post trial baseline follow-up** |
| --- | --- | --- | --- |
| Patient basic SES characteristics | Yes | Yes | Yes |
| Death | Yes | Yes | Yes |
| EQ5D | Yes | Yes | Yes |
| Patients’ medicine taking and adherence on Aspirin, Statin, Anti-hypertensive drugs, including the type of medicine they are taking, the continuation of medicine taking in the past year and the continuation of medicine taking in the past 14 days | Yes | Yes | Yes |
| Inpatient records only related to stroke (in the past one year) | Yes ^a^ | Yes ^b^ | Yes ^a^ |
| All-cause inpatient records (in the past one year) | Yes ^a^ | Yes ^b^ | No |
| Outpatient records | No | No | No |

SES: Socioeconomic Status; MRS: Modified Rankin Scale; EQ5D: EuroQol five dimensions questionnaire

a Indicates information gathered via questionnaires, independent of administrative data.

b Data obtained from the health insurance system's administrative records of local hospitals.

## **Table S4: Detailed Method for 6-year post-trial baseline effectiveness and cost calculation**

|  | Detailed method |
| --- | --- |
| Quality Adjusted Life Years (QALYs) | Health utility scores for the intervention group were estimated using a linear imputation method based on the 1-year follow-up and 6-year post-trial baseline follow-up data. For patients who died, the utility score was assigned as 0. QALYs were calculated annually using the area under the curve method and using the 5% discount rate. |
| Inpatient costs | For the post-trial period, we did not have access to inpatient records from the insurance system. Inpatient costs were calculated based on the categorized into three groups: stroke-related, CVD-related (excluding stroke), and all other causes.  For stroke-related inpatient costs, our post-trial survey included a specific question about hospitalizations due to stroke in the preceding post trial baseline-6 years. Cumulative costs were estimated by multiplying the average inpatient costs for the intervention or control group by the total number of stroke-related hospitalizations and adjusting for the time value of money using 5% discount rate.  For CVD-related and other inpatient costs, we first used data from the post-trial follow-up to calculate the costs incurred in the past year (2021-2022). We then used linear imputation method based on the costs from the 1-year (2017-2018) and 5-year follow-up periods (2021-2022) to estimate the cumulative costs over the 5 years. Specifically, if a patient's inpatient costs at the 6-year follow-up are higher than those at the 1-year follow-up, we interpret this as an increasing linear trend. Conversely, if the costs are lower, we assume a decreasing linear trend. If the costs remain relatively stable between the two follow-ups, we consider this a constant cost trend. The discount rate is 5%. |
| Outpatient costs | Like the within-trial period, we calculated outpatient costs based on data from relevant literature. The discount rate is 5%. |
| Medication costs | For patients who continued their medication at the 6-year post-trial baseline follow-up, we applied a linear imputation method to estimate medication costs. The drug costs were collected at consistent time points after the trial to avoid the costs may change over time. This method assumes a linear trend in costs over time. Specifically, if a patient's medication costs at the 5.5-year follow-up are higher than those at the 1-year follow-up, we interpret this as an increasing linear trend. Conversely, if the costs are lower, we assume a decreasing linear trend. If the costs remain relatively stable between the two follow-ups, we consider this a constant cost trend. . The discount rate is 5%. |
| Intervention costs | Incurred solely during the trial period. |

## **Table S5: Scale-up population and cost estimation methodology**

| **Category** | **Year 1** | | **Years 2-5** |
| --- | --- | --- | --- |
| **Scenario A: Standalone project** | | | |
| **Eligible population** | | To estimate the number of people expected to receive SINEMA in the first year of scale-up, we multiplied estimates of the rural population in China by stroke prevalence rates, including rates obtained during screening visits at our trial sites.  **Total population of China**: 1412000000  **Prevalence of stroke**: 2.58%  **Stroke Participant percentage in rural places**: 52.90%  **Screening rate collected in the trial for eligible participants**: 60.59% | For subsequent years, we estimated cases based on an annual stroke incidence rate of 8.26%, assuming a 1% annual national population growth and a 0.16% death rate. |
| **Training for village doctors (including compensation for county-level experts providing training and support, and printing materials)** | | Three routine training sessions will be conducted annually to ensure all village doctors can participate.  Each training session will last for half a day, totaling 1.5 days of training per year per doctor.  Training will be conducted in 2,843 county-level areas.  The training costs include materials and compensation for the trainers. The training costs are calculated by multiplying the average cost per county by the total number of counties. | Subsequent annual training will be reduced to a total of 0.5 days per year, with the training costs reduced to one-third of the first-year cost.  Cost reduction accounts for the shorter training duration and decreased labor demand from county-level doctors[1, 2]. |
| **Compensation for township doctor supervision** | | The supervision cost is calculated by multiplying the cost per participant by the total number of eligible participants each year. | The subsequent annual cost is reduced to 50% of the first-year cost to account for the decreased labor demand for supervision[1, 2]. |
| **Compensation for local project manager coordination** | | The coordination cost is calculated by multiplying the cost per participant by the total number of eligible participants each year. | The cost in subsequent years is reduced to 50% of the first-year cost to account for the decreased demand for coordination[1, 2]. |
| **Compensation for village doctors** | | The compensation is calculated based on the frequency of patient visits, with a subsidy of 5 RMB per visit. In the first year, there are 12 visits. | The subsequent annual visit frequency is reduced by one thirds each year based on the previous year's visit frequency。  The frequency will be reduced to at least four times per year, in line with national basic public health requirements, with no further reductions. |
| **Annual voice messages** | | Voice messages are sent daily to eligible participants. | Voice messages are sent daily to participants who experienced a new stroke that year. The frequency of messages sent to the same participants is reduced by 50% each year. |
| **Annual system maintenance and management costs** | | Collected during the trial period. | Same as the first year. |
| **Intervention APP development costs** | | Collected during the trial period, only calculated for the first year. | Not included in subsequent years. |
| **Medication costs** | | Use the collected trial data to analyze medication usage rates and changes in medication adherence between the intervention and control groups.  In China, the government covers approximately 80% of the costs for medications listed in Category B of the Essential Medicines List. Therefore, we assumed that 80% of the costs for the two types of medications were covered by the government.  The drug usage rate is collected from our trial and adopted to the eligible population. | Same as the first year |
| **Scenario B: Integrated into national basic public health services** | | | |
| **Eligible population** | | As the national basic public health services currently cover only patients with hypertension and diabetes, we categorized stroke patients into two groups: those with hypertension or diabetes who are included in the national basic public health services, and those not receiving the national basic public health services. | Same as the first year. |
| **Training for village doctors, compensation for township doctor supervision, compensation for local project manager coordination** | | The three components will be integrated with current national basic public health services, which will not include in the cost estimation | Same as the first year. |
| **Compensation for village doctors** | | For patients receiving national basic public health services, an additional 8 visits in the first year were needed, as they already receive quarterly visits from village doctors. For patients not covered by the national basic public health services, 12 visits were required. | The subsequent annual visit frequency is reduced by one thirds each year based on the previous year's visit frequency。  The frequency will be reduced to at least four times per year, in line with basic national public health requirements, with no further reductions.  The frequency is calculated same as the first year. |
| **Annual Voice Messages** | | Same as the scenario A | Same as the scenario A |
| **Intervention Development and Maintenance System** | | Since the national basic public health services has already developed and updated a system to record eligible participants’ blood pressure and remind village doctors to conduct regular follow-ups, we assumed that their system could perform the same functions as the SINEMA app. Therefore, we did not consider the system costs in this scenario. | Same as the first year. |
| **Medication Costs** | | Same as the scenario A | Same as the scenario A |

## **Table S6: Impact of the SINEMA Intervention on Health Outcomes**

|  | **Baseline** | | **Change at 12 months from baseline** | | **Difference in change between Intervention and Control^a^** |
| --- | --- | --- | --- | --- | --- |
|  | **Intervention**  **(n=637)** | **Control**  **(n=662)** | **Intervention**  **(n=611)** | **Control**  **(n=615)** |  |
| **Total Antihypertensive Medications Used with Days of Use^b^** | 10.71 (5.73) | 10.15 (6.10) | 11.37 (5.22) | 9.98 (6.04) | -0.87 (-1.41, -0.03) |
| **Total Statin Medications Used with Days of Use^b^** | 3.20 (5.82) | 3.42 (5.82) | 3.71 (6.09) | 3.66 (6.03) | -0.47 (-1.15, 0.21) |
| **Total Antiplatelet Medications Used with Days of Use^b^** | 8.98 (6.60) | 8.38 (6.75) | 9.55 (6.30) | 7.80 (6.76) | -0.96 (-1.64, -0.28) |
| **Hospitalized due to stroke (past 12 months)^c^** | 124 (19.5%) | 132 (19.9%) | 29 (4.7%) | 46 (7.5%) | -0.03 (-0.30, 0.24) |

a Difference is adjusted for baseline outcome, township, sex, and age.

b Three types of medications were quantified by multiplying the number of days the medications were taken by whether they were used within the past 14 days, expressed in person*days.

c This variable indicates whether a participant was hospitalized due to stroke during the past 12 months.

## **Table S7: Characteristics of participants at baseline refer to the work from Yan et al. ^a^**

| **Characteristics** | **Study Arms** | | **Total**  **(n=1299)** |
| --- | --- | --- | --- |
|  | **Intervention**  **(n=637)** | **Control**  **(n=662)** |  |
| **Age, years** | 65.2 (8.2) | 66.2 (8.2) | 65.7 (8.2) |
| **Sex, % female** | 281 (42.4%) | 272 (42.7%) | 553 (42.6%) |
| **Education** |  |  |  |
| Haven't been to school | 515 (42.0%) | 23 (31.5%) | 538 (41.4%) |
| Primary school | 359 (29.3%) | 28 (38.4%) | 387 (29.8%) |
| Primary high school and above | 352 (28.7%) | 22 (30.1%) | 374 (28.8%) |
| **Stroke type** |  |  |  |
| Ischemic | 564 (85.2%) | 555 (87.1%) | 1,119 (86.1%) |
| Hemorrhage | 96 (14.5%) | 80 (12.6%) | 176 (13.6%) |
| Not specified | 2 (0.3%) | 2 (0.3%) | 4(0.3%) |
| **Taking medicines** |  |  |  |
| Antiplatelet | 420 (63.4%) | 432 (67.8%) | 852 (65.6%) |
| Statin | 182 (27.5%) | 158 (24.8%) | 340 (26.2%) |
| Anti-hypertensive medicines | 508 (76.7%) | 522 (81.9%) | 1,030 (79.3%) |
| **Adherent to medicine taking** |  |  |  |
| Antiplatelet | 262 (62.4%) | 275 (63.7%) | 537 (63.0%) |
| Statin | 110 (60.4%) | 106 (67.1%) | 216 (63.5%) |
| Anti-hypertensive medicines | 262 (62.4%) | 275 (63.7%) | 537 (63.0%) |
| **Systolic blood pressure at baseline, mmHg** | 146.0 (20.9) | 145.7 (23.7) | 145.9 (22.4) |
| **Diastolic Blood Pressure at baseline, mmHg** | 78.0 (11.6) | 79.7 (11.7) | 78.9 (11.7) |
| **Health-related quality of life score at baseline** | 0.80 (0.2) | 0.8 (0.21) | 0.8 (0.2) |

Data are mean (standard deviation), n(%)

**a** This table includes data partially extracted from our previously published paper: Yan, L. L., Gong, E., Gu, W., Turner, E. L., Gallis, J. A., Zhou, Y., Li, Z., McCormack, K. E., Xu, L. Q., Bettger, J. P., Tang, S., Wang, Y., & Oldenburg, B. (2021). Effectiveness of a primary care-based integrated mobile health intervention for stroke management in rural China (SINEMA): A cluster-randomized controlled trial. PLoS medicine, 18(4), e1003582. https://doi.org/10.1371/journal.pmed.1003582

## **Table S8: Analysis of Loss to Follow-Up Differences Between Groups Within and Post Trial ^a^**

|  | **Within Trial** | | | **Post Trial** | | |
| --- | --- | --- | --- | --- | --- | --- |
|  | **Intervention** | **Control** | **Mean difference (95%CI) or P value ^c^** | **Intervention** | **Control** | **Mean difference (95%CI) or P value ^c^** |
| **Inclusion**^b^ | 622 (97.6%) | 634(95.8%) | 0.52 (0.28, 0.96) | 600 (95.9%) | 625(97.2%) | 0.78 (0.64, 0.95) |
| Survival | 611(98.3%) | 615 (97.1%) |  | 490 (81.7%) | 489 (78.2%) |  |
| Died | 11(1.7%) | 19 (2.9%) |  | 110 (18.3%) | 136 (21.8%) |  |
| **Exclusion**^b^ | 15(2.4%) | 28(4.2%) | 0.06 | 28(4.2%) | 18(2.8%) | 0.19 |

Data is n (%)

a "Within trial period" pertains to the duration of the intervention, spanning from 2017 to 2018. "Post trial period" denotes the time following the conclusion of the intervention, commencing after 2018.

b Inclusion refers to participants who survived or died during the study. Exclusion refers to those who lost to follow-up during each time period, who were therefore excluded from the economic evaluation.

c For each period, death and loss to follow-up rates were tested between the two arms. Mean differences represent the death rate differences, while the loss to follow-up rates is reported as P-values.

## **Table S9: Budget impact in China in years 1–5 from the health ministry perspective**

|  | **Year 1** | **Year 2** | **Year 3** | **Year 4** | **Year 5** |
| --- | --- | --- | --- | --- | --- |
| **Scenario A: Standalone project** | | | | | |
| Training costs^a^ | $382,952.11 | $127,650.70 | $127,650.70 | $127,650.70 | $127,650.70 |
| Compensation for township physicians' oversight work | $21,016,817.35 | $10,512,717.12 | $10,512,717.12 | $10,512,717.12 | $10,512,717.12 |
| Compensation for the local project manager’s coordination work | $14,011,211.56 | $7,008,478.08 | $7,008,478.08 | $7,008,478.08 | $7,008,478.08 |
| Compensation for village doctors | $97,707,193.61 | $73,310,440.17 | $48,893,664.97 | $32,609,140.91 | $32,622,510.66 |
| Voice messages yearly | $28,022,423.13 | $12,907,301.54 | $6,482,188.42 | $3,268,325.33 | $1,660,745.57 |
| System maintenance and management costs per year | $26,854,822.16 | $14,045,118.70 | $7,053,612.68 | $3,556,437.96 | $1,807,145.24 |
| Intervention APP development fee | $16,497.62 | $0 | $0 | $0 | $0 |
| Antiplatelet drugs | $7,565,120.16 | $7,568,221.86 | $7,571,324.83 | $7,574,429.08 | $7,577,534.59 |
| Anti-hypertension drugs | $2,149,319.85 | $2,150,201.08 | $2,151,082.66 | $2,151,964.60 | $2,152,846.91 |
| Cost per participant | $16.93 | $11.21 | $8.52 | $6.83 | $6.68 |
| Cost per capita^b^ | $0.14 | $0.09 | $0.07 | $0.06 | $0.06 |
| Eligible population | 11,676,010 | 11,680,797 | 11,685,586 | 11,690,377 | 11,695,170 |
| Total budget impact | $197,726,357 | $130,948,292 | $99,555,011 | $79,788,318 | $78,067,407 |
| **Scenario B: Integrated with national basic public health** | | | | | |
| Training costs^a^, compensation for township physicians, local project manager | $0 | $0 | $0 | $0 | $0 |
| Compensation for village doctors | $77,832,275.50 | $53,427,373.34 | $29,849,061.43 | $8,152,285.23 | $8,155,627.67 |
| Voice messages yearly | $28,022,423.13 | $12,907,301.54 | $6,482,188.42 | $3,268,325.33 | $1,660,745.57 |
| Intervention APP development fee and maintenance fee | $0 | $0 | $0 | $0 | $0 |
| Cost per participant | $9.90 | $8.91 | $6.29 | $4.13 | $3.98 |
| Cost per capita^b^ | $0.08 | $0.07 | $0.05 | $0.03 | $0.03 |
| Eligible Participants receiving NBPH† | 7,125,158 | 7,128,079 | 7,131,002 | 7,133,926 | 7,136,851 |
| Eligible Participants excluded from NBPH^c^ | 4,550,852 | 4,552,717 | 4,554,584 | 4,556,451 | 4,558,319 |
| Total budget impact | $115,585,636 | $104,056,748 | $73,501,929 | $48,322,984 | $46,592,046 |

Data are presented in 2022 US dollars or as counts (n). Costs are rounded to the nearest $0.01, while total budget impacts are rounded to the nearest $10.

a Training costs include compensation for county specialists and training materials printing fee.

b Cost per capita is calculated as the total cost divided by the national population.

c Eligible participants receiving NBPH are individuals with stroke and hypertension (or diabetes) living in rural communities. Eligible participants excluded from NBPH are individuals with stroke who do not have hypertension (or diabetes).

## **Supplementary references**

**S1.** Yan, L. L., Gong, E., Gu, W., Turner, E. L., Gallis, J. A., Zhou, Y., Li, Z., McCormack, K. E., Xu, L. Q., Bettger, J. P., Tang, S., Wang, Y., & Oldenburg, B. (2021). Effectiveness of a primary care-based integrated mobile health intervention for stroke management in rural China (SINEMA): A cluster-randomized controlled trial. *PLOS Medicine, 18*(4), e1003582. Available at: https://doi.org/10.1371/journal.pmed.1003582.

**S2.** Zhang, Y., Yin, L., Mills, K., Chen, J., He, J., Palacios, A., Riviere, A. P., Irazola, V., Augustovski, F., & Shi, L. (2021). Cost-effectiveness of a multicomponent intervention for hypertension control in low-income settings in Argentina. *JAMA Network Open, 4*(9), e2122559. doi: 10.1001/jamanetworkopen.2021.22559. PMID: 34519769; PMCID: PMC8441594.

**S3.** Chen, Y., Wright, N., Guo, Y., et al. (2020). Mortality and recurrent vascular events after first incident stroke: A 9-year community-based study of 0.5 million Chinese adults. *The Lancet Global Health, 8*(4), e580-e590.

**S4.** Tu, W. J., & Wang, L. D. (2023). China stroke surveillance report 2021. *Military Medical Research, 10*(1), 33.

**S5.** National Health Commission. (2022). *China Health Statistical Yearbook 2022*. China Union Medical University Press: Beijing, China.

**S6.** You, L., & Liu, Y. (2022). National Essential Public Health Services Programs over the Past Decade: Research Report One - Significance and Experiences of Implementing National Essential Public Health Services Programs. *Chinese General Practice, 25*(26), 3203-3208. DOI: 10.12114/j.issn.1007-9572.2022.0441.

1. Finkelstein EA, Krishnan A, Naheed A, Jehan I, De Silva HA, Gandhi M, et al. Budget impact and cost-effectiveness analyses of the COBRA-BPS multicomponent hypertension management programme in rural communities in Bangladesh, Pakistan, and Sri Lanka. The Lancet Global Health. 2021;9(5):e660-e7. doi: 10.1016/s2214-109x(21)00033-4.

2. Krishnan A, Finkelstein EA, Kallestrup P, Karki A, Olsen MH, Neupane D. Cost-effectiveness and budget impact of the community-based management of hypertension in Nepal study (COBIN): a retrospective analysis. The Lancet Global Health. 2019;7(10):e1367-e74. doi: 10.1016/s2214-109x(19)30338-9.
